# Nanoliposome-Mediated Encapsulation of Chlorella Oil for the Development of a Controlled-Release Lipid-Lowering Formulation

**DOI:** 10.3390/foods13010158

**Published:** 2024-01-02

**Authors:** Lanlan Tu, Jihao Zeng, Xue Bai, Ziyun Wu, Jinhong Wu, Shannan Xu

**Affiliations:** 1 Department of Food Science and Engineering, School of Agriculture and Biology, Shanghai Jiao Tong University, Shanghai 200240, China; tull@gempharmatech.edu (L.T.); 1811091098@nbu.edu.cn (J.Z.); baixueff@sjtu.edu.cn (X.B.); wuziyun@sjtu.edu.cn (Z.W.); 2 South China Sea Fisheries Research Institute, Chinese Academy of Fishery Sciences, Guangzhou 510300, China

**Keywords:** chlorella oil, nanoliposome, hypolipidemic activity, in vitro digestion simulation

## Abstract

Chlorella oil nanoliposomes (CO-NLP) were synthesized through ultrasonic injection with ethanol, and their physicochemical properties and hypolipidemic efficacy were systematically investigated. The results revealed that the mean particle size of CO-NLP was 86.90 nm and the encapsulation efficiency (EE) was 92.84%. Storage conditions at 4 °C were conducive to the stability of CO-NLP, maintaining an EE of approximately 90% even after 10 days of storage. The release profile of CO-NLP adhered more closely to the first-order kinetic model during in vitro assessments, exhibiting a slower release rate compared to free microalgae oil. In simulated in vitro digestion experiments, lipolytic reactions of CO-NLP were observed during intestinal digestion subsequent to nanoliposome administration. Notably, the inhibitory effect of CO-NLP on cholesterol esterase activity was measured at 85.42%. Additionally, the average fluorescence intensity of nematodes in the CO-NLP group was 52.17% lower than in the control group at a CO-NLP concentration of 500 μg/mL, which suggests a pronounced lipid-lowering effect of CO-NLP. Therefore, the CO-NLP exhibited characteristics of small and uniform particle size, elevated storage stability, gradual release during intestinal digestion, and a noteworthy hypolipidemic effect. These findings designate CO-NLP as a novel lipid-lowering active product, demonstrating potential for the development of functional foods.

## 1. Introduction

Microalgae oil, specifically chlorella oil, contains linoleic acid, γ-linolenic acid, and various other fatty acids with substantial effects on reducing blood pressure, blood glucose, and lipids. Additionally, it is abundant in unsaturated fatty acids, including docosahexaenoic acid (DHA), referred to as “brain gold”, promoting the growth and development of brain cells. This makes microalgae oil widely utilized in health products and infant formula [1]. While it is the only FDA-approved source of DHA supplements for children, challenges exist in its application [2]. Firstly, due to the unsaturation of fatty acids and poor oxidative stability, microalgae oil not only loses its original nutritional value post-oxidation but also produces harmful oxidation by-products. Secondly, microalgae oil, being a highly hydrophobic molecule with low water solubility, cannot be easily incorporated into most water-based foods and beverages. Finally, microalgae oil often undergoes structural and functional degradation in the acidic gastric environment during digestion, leading to a significant reduction in in vivo bioavailability.

Nanoliposomes, vesicular lipid bilayer nanocarriers with a spherical shape typically composed of natural, non-toxic phospholipids and cholesterol, present a viable solution. The advantage of liposomes over nanometer emulsions and microcapsules lies in their ability to form a system carrying both oil and water [3]. This enables the encapsulation of both lipophilic and hydrophilic substances in nanoliposomes, resulting in a larger surface-to-volume ratio. Nanoliposomes have found extensive use in functional foods, enhancing nutrient solubility, delaying release, and maintaining substance activity due to their high safety, non-immunological reactivity, biocompatibility, and biodegradability [4]. Research indicates nanoliposomes as suitable encapsulants for entrapping and delivering various vitamins to target cells, enhancing their bioaccessibility and bioavailability [5]. Loading polyphenol-rich herbal extract into nanoliposomes demonstrated significant glucose-lowering activity in vivo compared to free extract [6]. Liposomes produced from 4% phospholipids and loaded with astaxanthin exhibited high antioxidant activity [7], with astaxanthin encapsulated in nanoliposomes achieving an 80.31% high encapsulation efficiency [8]. In the development of fortified bread as a functional food, fish oil nanoliposomes did not adversely affect the texture, nutritional content, or sensory qualities of the fortified bread [9].

To enhance the application stability and bioavailability of chlorella oil, this study opted for a specific small unilamellar vesicle nanoliposome to encapsulate it. The investigation delves into physicochemical properties, morphological characteristics, release kinetics, stability, and lipid-lowering activity. This study aimed to develop a novel functional chlorella oil carrier, enhancing solubility, improving bioavailability, and achieving better-controlled release of the encapsulated material. This study provides the theoretical basis and technical support for the industrial production of functional chlorella oil.

## 2. Materials and Methods

### 2.1. Reagents

All chemicals employed in this study, including the culture medium, trichloroacetic acid, soy lecithin, β-sitosterol, trypsin, porcine pancreatic cholesterol esterase, phosphate salts, and boron trifluoride, were sourced from Sigma Aldrich (Shanghai) Trading Co., Ltd., Shanghai, China. Tween-80, sodium hydroxide, sodium chloride, n-heptane, anhydrous sodium sulfate, Triton X-100, methanol, phosphotungstic acid, p-nitrophenyl butyrate, taurocholate buffer and imidazole were purchased from Sinopharm Chemical Reagent Co., Ltd., Shanghai, China. The reagents utilized were of ACS grade. The preparation of all solutions was executed utilizing purified and deionized ultra-pure water.

### 2.2. Preparation of CO-NLP

Ultrasonic injection with ethanol was employed in the preparation of CO-NLP in this study. Prior investigations explored the impact of various technological factors, including the mass ratio of lecithin to β-sitosterol, the mass ratio of lecithin to chlorella oil, the addition of Tween-80, the pH of PBS buffer, ultrasonic time, and ultrasonic power, on key parameters such as encapsulation efficiency (EE), particle size, and the PDI. These investigations encompassed single-factor experiments, Plackett-Burman experiments, and Box-Behnken experiments. Optimal preparation conditions were identified as follows: a lecithin concentration of 10 mg/mL, a lecithin to β-sitosterol mass ratio of 5.26:1 (*w/w*), a lecithin to chlorella oil mass ratio of 6:1 (*w/w*), a Tween-80 concentration of 8.18%, a pH of 7.0, an ultrasonic time of 9 min, and an ultrasonic power of 264 W (refer to supplementary experimental results in the Supplementary Materials). Therefore, the preparation of CO-NLP involved the agitated dissolution of lecithin, β-sitosterol, Tween-80, and chlorella oil in ethanol at 55 °C in a water bath for 30 min, adhering to the optimal preparation conditions. Following complete dissolution, the resultant mixture was swiftly injected into pH 7.0, 0.05 mol/L PBS buffer using a sterile syringe, and stirred at 55 °C for 30 min, with the stirring rate set at 100 r/min. Subsequent to this, the ethanol underwent rotary evaporation under a reduced pressure of 0.1 MPa at 40 °C, yielding crude chlorella oil liposomes. This product then underwent ultrasonication under an ultrasonic power of 264 W for 9 min via an ultrasonic cell crusher (JY92-HD, Shanghai Bilang Instrument Co., Ltd., Shanghai, China) to generate nanoliposomes, which were then stored in a refrigerator at 4 °C for further analysis.

### 2.3. Analysis of Chlorella Oil Content

The soy lecithin, β-sitosterol, and chlorella oil were dissolved in a solution of 0.20 mg/mL petroleum ether. The absorbance of each sample within the range of 190 to 400 nm was measured using a UV absorbance photometer with petroleum ether as a blank. The optimal absorption wavelength of chlorella oil was determined by comparing it to the other samples. Subsequently, chlorella oil concentrations of 0.01, 0.02, 0.05, 0.10, and 0.15 mg/mL were prepared utilizing petroleum ether as the solvent since both soy lecithin and chlorella oil are soluble in petroleum ether. A standard curve of chlorella oil was then graphed at the optimal absorption wavelength for the purpose of content analysis.

### 2.4. Analysis of the Fatty Acid Composition of Chlorella Oil

The fatty acid composition of chlorella oil was determined using a high-performance GC instrument (GC 2010 Plus, Shimadzu, Japan). In a flask containing chlorella oil, 8 mL of a 2% sodium hydroxide in methanol solution was added, and reflux condensation was performed at 80 ± 1 °C until the oil droplets disappeared. Subsequently, 7 mL of a 15% boron trifluoride in methanol solution was introduced into the flask, and the solution was further refluxed for 2 min in an 80 ± 1 °C water bath. The flask was then removed from the water bath and quickly cooled to room temperature. Accurately measured quantities of 10 mL to 30 mL of n-heptane were added to the flask, and the mixture was shaken for 2 min. Then, a saturated sodium chloride aqueous solution was added, and the solution was allowed to stand for stratification. From the upper extraction solution with n-heptane, 5 mL was added into a 25 mL test tube. Following this, 3 g to 5 g of anhydrous sodium sulfate was added, the solution was shaken for 1 min, allowed to stand for 5 min, and the upper solution was absorbed into the injection bottle for subsequent analysis.

For the GC analysis of fatty acid composition, the specified conditions were aligned with China’s national testing standard [10]. These included: a capillary chromatography column with a robust polar stationary phase of polydicyanopropyl siloxane, measuring 100 mm in length, 0.25 mm in inner diameter, and 0.2 μm in membrane thickness; an injector temperature of 270 °C for the sample; a detector temperature of 280 °C; a carrier gas to helium gas ratio; a split ratio of 100:1; and an injection and an injection volume of 1.0 μL. The heating protocol involved starting at 100 °C for 13 min, a heating rate of 100 °C to 180 °C for 6 min, a rate of 180 °C to 200 °C per minute for 20 min, and a heating rate of 200 °C to 230 °C for 10.5 min. It is essential that the detection parameters align with the theoretical trays (n) minimum of 2000 pieces/m, and the separation degree (R) should be no less than 1.25.

### 2.5. Encapsulation Efficiency

The encapsulation efficiency (EE) of chlorella oil was determined according to Wang’s method [11], with slight modifications. A 10 mL volumetric flask was utilized, into which 1 mL of CO-NLP and 1 mL of a 10% Triton X-100 methanol solution, acting as an emulsion breaker, were added. Emulsion was broken by ultrasonication for 10 min, followed by vortex shaking to ensure complete liposome disintegration. The mixture was then centrifuged at 4000 rpm for 10 min. The quantification of free chlorella oil content in the supernatant was carried out based on the standard curve of chlorella oil.

The EE was calculated using the following Equation (1):

(1) $$\mathrm{Encapsulation}\;\mathrm{efficiency}\;\left(\mathrm{EE}\right)\%=\left(1-\frac{m_{\mathrm{free}}}{m_{\mathrm{total}}}\right)\times 100$$

In the formula, m_free_ represents the content of free chlorella oil in the supernatant, and m_total_ represents the content of total chlorella oil in the supernatant.

### 2.6. Determination of Particle Size, Zeta Potential, and Polydispersity Coefficient

The measured sample was introduced into a transparent cuvette with a refractive index of 1.33 and maintained at 25.0 ± 0.1 °C for 3 min. Following this, the nanometer-sized zeta potentiometer (Nano-ZS90, Malvern Instruments Limited, Malvern, UK) was employed to assess the average particle size, zeta potential, and particle size distribution (polymer dispersibility index, PDI). The measurements for each sample were conducted simultaneously in triplicate.

### 2.7. Microscopic Morphological Analysis

The CO-NLP was dispersed in water and then applied to a copper grid for drying. Following excess liquid absorption by filter paper, it underwent immersion in a 2% phosphotungstic acid solution for staining, followed by drying, and the microscopic morphological structure of CO-NLP was observed using Transmission Electron Microscopy (TEM) [12].

### 2.8. Storage Stability of CO-NLP

The stability of CO-NLP was evaluated by measuring its EE, particle size, PDI, and zeta potential over durations of 5 d, 10 d, 20 d, 40 d, and 60 d at temperatures of 4 °C and 25 °C, respectively.

### 2.9. In Vitro Release Experiments of CO-NLP

A pretreated dialysis bag (with a molecular weight cutoff of 12,000 Da) was filled with 10 mL of CO-NLP using a pipette, securely fastened, and submerged in 200 mL of PBS buffer (pH = 7.4) containing 1% Tween-80. The samples were placed on a magnetic stirrer, rotating at a rate of 100 r/min at a temperature of 37 ± 1 °C.

Subsequently, 10 mL of solution was collected at intervals of 0.5, 1, 2, 4, 6, 8, 10, 12, 24, 36, and 48 h for measurement. The PBS buffer release medium was promptly replenished at the same temperature after each sampling to ensure a constant total volume.

The cumulative release rate (Q%) was calculated using the Formula (2):

(2) $$\mathrm{Cumulative}\;\mathrm{release}\;\mathrm{rate}\;Q\left(\%\right)=\frac{V_{e}\sum_{i=1}^{t-1}C_{i}+V_{0}C_{t}}{m_{0}}\times 100$$

where V_e_ is the sampling volume (10 mL), V_0_ is the total volume (200 mL), C_i_ and C_t_ are chlorella oil concentrations in the solution at different release times (mg/L), and m_0_ is the mass of encapsulated oil in liposomes (mg).

Four release kinetic equations were selected for release analysis: the zero-order kinetic equation, the first-order kinetic equation, the Higuchi plane diffusion equation, and the Retger-Peppas equation. The curves were fitted to evaluate the accuracy of the fit, and then the in vitro release type of CO-NLP was determined to elucidate the pattern of chlorella oil liposome release.

### 2.10. In Vitro Digestion Experiments

An in vitro-simulated gastrointestinal tract digestion model was established following the experimental protocol of Xu et al. [13]. This model comprised the oral stage, gastric stage, and intestinal stage. The assessment of sample conditions during digestion involved the measurement of particle size and zeta potential at each stage.

### 2.11. In Vitro Simulated Digestion Bioavailability Study

To maintain a pH of 7.0 during the simulated small intestine stage, a 0.25 mol/L NaOH solution was incrementally added, and the consumption of NaOH was recorded at various time intervals to assess the level of chlorella oil digestion. The rate of release of free fatty acids (FFAs) was evaluated at 10 min intervals upon the addition of trypsin solution in order to simulate the digestive environment of the gastrointestinal tract, as liposomes can break down, emit free fatty acids, and lower the pH value of the environment in the presence of trypsin. Each digestion stage was terminated by transferring the digest to a glass tube and subjecting it to agitation at 85 °C for 5 min, followed by cooling in an ice water bath. The amount of NaOH consumed served as a measure of the rate of FFA release [4].

The rate of fat release (FFA)% was determined using the Formula (3):

(3) $$\mathrm{Rate}\;\mathrm{of}\;\mathrm{fat}\;\mathrm{release}\;\left(\mathrm{FFA}\right)\%=\frac{V_{\mathrm{NaOH}}\times M_{\mathrm{NaOH}}\times M_{\mathrm{Lipid}}}{2m_{\mathrm{Lipid}}}\times 100\times 10^{-3}$$

where M_NaOH_ is the concentration of NaOH solution (0.25 mol/L), V_NaOH_ is the volume of NaOH solution used (L), M_Lipid_ is the average molecular weight of fat molecules (g/mol), and its value is 923.08 g/mol, and m_Lipid_ is the total mass of fat in liposomes (g).

### 2.12. Calculation of the Particle Dimensions and Zeta Potential of Digestion Byproducts from CO-NLP

Prior to sampling, dilution of the sample was conducted in a suitable aqueous solution to mitigate any scattering effects. The system was then stirred at 1200 r/min to ensure consistency. Subsequently, the mean particle size and zeta potential in both gastric and intestinal digests were determined utilizing a nanoparticle size and zeta potential analyzer.

### 2.13. Assessment of Inhibitory Effects of CO-NLP on Porcine Pancreatic Cholesterol Esterase Activity

The approach proposed by Su [14] was employed with minor modifications. In brief, the measurement was conducted in a solution of 0.1 mol/L sodium phosphate (pH 7.04) containing 0.1 mol/L NaCl, 0.2 mmol/L p-nitrophenyl butyrate (PNPB), and 5.16 mmol/L sodium taurocholate buffer (STC). Initially, 10 U/mL of porcine pancreatic cholesterol esterase and PNPB were dissolved in acetonitrile and stored at −20 °C. The reaction tube was supplemented with porcine pancreatic cholesterol esterase, and the CO-NLP was allowed to incubate at 25 °C for 5 min. The absorbance of the solution was then measured at 450 nm using an ultraviolet-visible spectrophotometer. Table 1 details the additives and their respective concentrations in each tube. The calculation of percent inhibition utilized Equation (4):

(4) $$\mathrm{Inhibition}\;\mathrm{activity}\;\left(\%\right)=\left(1-\frac{A_{3}-A_{4}\;}{A_{1}-A_{2}}\right)\times 100$$

Where A_1_ is the absorbance of the blank group (with the sample solution substituted with an equal volume of buffer), A_2_ is the absorbance of the blank control group (with both the sample solution and enzyme solution substituted with an equal volume of buffer), A_3_ is the absorbance of the sample group, and A_4_ is the absorbance of the sample control group (with the enzyme solution substituted with an equal volume of buffer).

### 2.14. Assessment of the Hypolipidemic Impact of CO-NLP in the C. elegans Model

Initially, a solution of *Escherichia coli* (*E. coli*) with FEAK was prepared as follows: A mother liquor of CO-NLP at a concentration of 500 mg/mL was prepared and stored at −20 °C. Subsequently, 10 μL of the mother liquor was added to 10 mL of concentrated *E. coli* solution and mixed. The resulting mixture was applied to the nematode growth medium (NGM) agar plate and air-dried for future use. The control group received 10 μL of empty liposomes instead of CO-NLP. The impact of CO-NLP on lipid deposition was observed using the green fluorescence of the dhs-3:gfp mutants.

At the L1 stage, the animals were initially synchronized and then divided into groups. Approximately 50 nematodes were cultivated on each plate in a consistent temperature incubator, maintaining a temperature of 20 °C for 6 d. Subsequently, the animals were rinsed with M9 buffer, anesthetized with 40 mmol/L imidazole, and then examined and captured under a fluorescence microscope. Image J was employed to calculate the average mean fluorescence intensity.

### 2.15. Statistical Analysis

Three parallel groups were formed for each experimental group, and statistical results are presented as mean ± standard error (SE). Statistical analysis was performed using SPSS 26.0 software (IBM, 2022, Shanghai, China), with a significance level set at *p* < 0.05 to indicate statistical significance. GraphPad Prism 9.0 software (GraphPad Software, 2021, Shanghai, China) was utilized for generating graphical representations.

## 3. Results and Discussion

### 3.1. Analysis of Chlorella Oil Content and Fatty Acid Composition

**Figure 1:**
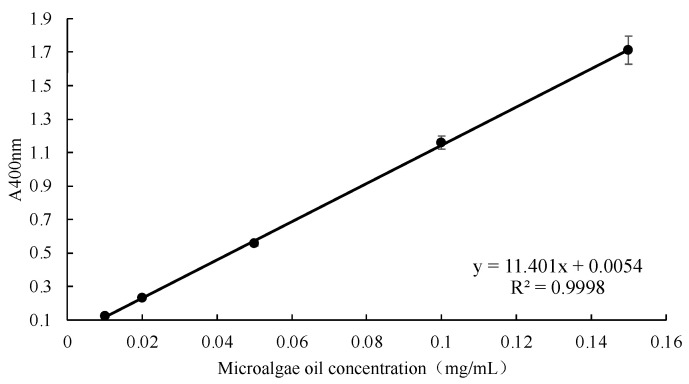
A standard curve is used for quantifying chlorella oil concentration.

The UV absorption spectrogram reveals the characteristic absorption wavelength of chlorella oil to be 400 nm, a wavelength strategically chosen for the quantification of chlorella oil content. Concentration gradients of 0.01, 0.02, 0.05, 0.10, and 0.15 mg/mL of the petroleum ether solution of chlorella oil were established, and absorbance measurements were conducted at the specified 400 nm wavelength. The resultant standard curve, as illustrated in Figure 1, was formulated with chlorella oil concentration as the horizontal coordinate (x) and absorbance values as the vertical coordinate (y). The derived equation, y = 11.4010x + 0.0054 (R^2^ = 0.9998), was subsequently employed for the precise quantification of chlorella oil.

Table 2 provides an in-depth analysis of the fatty acid composition in chlorella oil. The results highlight eicosapentaenoic acid (EPA) as the predominant unsaturated fatty acid present in the chlorella fatty acid extract, closely followed by the monounsaturated fatty acids palmitoleic acid and docosahexaenoic acid (DHA). Additionally, palmitic acid and myristicin were identified as the primary constituents in the sample. These findings unequivocally confirm the presence of unsaturated fatty acids (UFAs) in the chlorella oil extracts utilized in the current study, aligning with prior research [15].

### 3.2. Characterization of the Physical and Chemical Attributes of CO-NLP

To explicate the physicochemical characteristics of CO-NLP, this study focused on the systematic examination of its particle size, zeta potential, microscopic morphology, storage stability, and in vitro release characteristics, each addressed individually.

#### 3.2.1. Particle Size and Zeta Potential of CO-NLP

**Figure 2:**
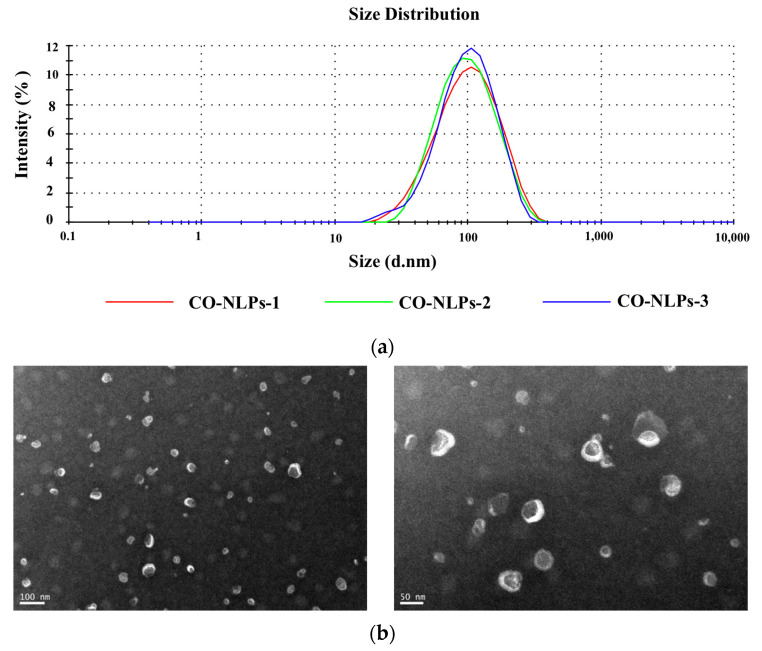
Diameter distribution (**a**) and electron micrographs illustrating the morphology (**b**) with a scale bar of 100 nm (**left**) and 50 nm (**right**) of CO-NLP.

The CO-NLP demonstrated a mean particle size of 86.90 nm, displaying a singular peak shape indicative of a normal distribution. This characteristic underscores the uniformity and robustness of the liposome system, as illustrated in Figure 2a. The particle size distribution further affirmed uniformity, supported by a low PDI of 0.19. The CO-NLP exhibited a negative charge, as evidenced by its zeta potential of −25.05 mV. Notably, a significant zeta potential, whether positive or negative, acts as a deterrent to the mutual aggregation of liposomes, thereby enhancing their stability to a certain extent [16].

Ultrasound-induced cavitation played a crucial role in the reduction and even distribution of liposome particles. Additionally, the membrane material of CO-NLP, soy phospholipid, carries a negative charge, fostering electrostatic repulsion between nanoliposomes and altering their surface charge. This phenomenon diminishes the likelihood of polymerization and contributes significantly to improved stability.

#### 3.2.2. Microscopic Morphological Observation

The morphology of CO-NLP was observed using Transmission Electron Microscopy (TEM), revealing predominantly rounded and spherical particles in the images. The particles exhibited homogeneous sizing and distinctive vesicle-like structures. They were dispersed without apparent connections, collapse, or clustering, with only a few irregular forms. Subsequent to ultrasonic treatment, chlorella oil liposomes exhibited a more uniform particle distribution, with sizes below 100 nm, as illustrated in Figure 2b.

The disparity in particle size outcomes between TEM and the particle size analyzer could be attributed to the liquid state of the microalgal oil measured by the particle size analyzer, representing hydrodynamic particle size and resulting in a larger measured size compared to TEM, which operates under the dehydrated composition of the sample [17]. Furthermore, the two measurement methods diverge in their underlying principles. Dynamic light scattering, the basis for particle size determination, relies on particle diffusion characteristics, with the system’s viscosity impacting the results [18].

#### 3.2.3. Analysis of the Storage Stability of CO-NLP

**Figure 3:**
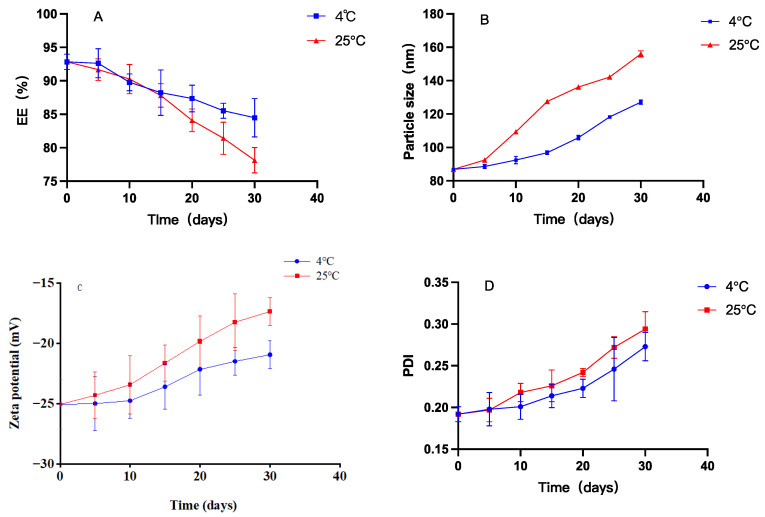
Storage stability assessment of CO-NLP over time, with panels depicting (**A**) EE, (**B**) Size, (**C**) Zeta Potential, and (**D**) PDI.

**Figure 4:**
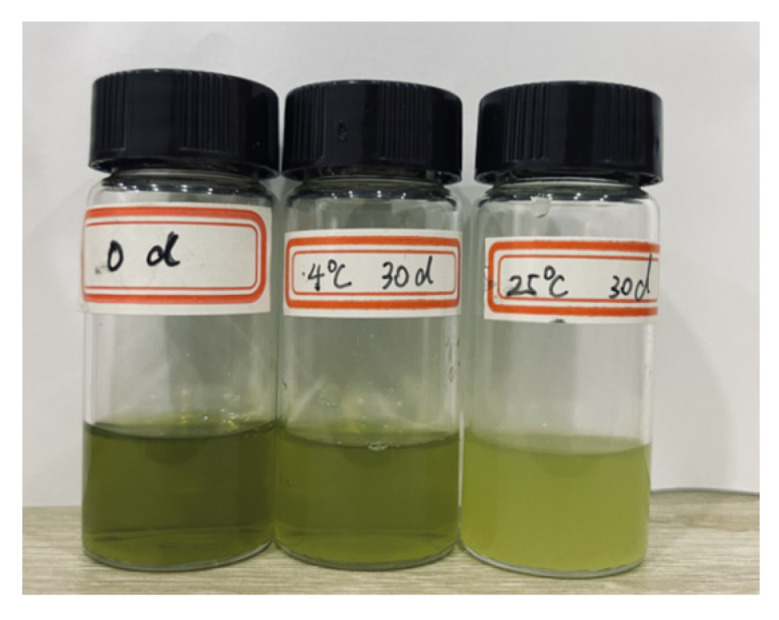
Preservation status of CO-NLP at various temperatures after a 30 d period.

Figure 3 illustrates variations in EE, particle size, zeta potential, and PDI of CO-NLP during storage at temperatures of 4 °C and 25 °C. The graph depicts a decrease in CO-NLP EE from 92.84 ± 1.14% to 84.48 ± 2.86%, an increase in particle size from 86.90 ± 0.74 nm to 127.14 ± 1.37 nm, a rise in PDI from 0.19 ± 0.01 to 0.27 ± 0.02, and a decline in zeta potential from −25.05 ± 2.07 mV to −20.93 ± 1.17 mV after 30 d of storage at 4 °C. Although the EE experienced a slight reduction below 4 °C, it remained around 90% after 10 d, indicating stable CO-NLP that effectively prevented oil leakage. At 25 °C, the EE of CO-NLP decreased from 92.84 ± 1.14% to 78.14 ± 1.91%, accompanied by an increase in particle size from 86.90 ± 0.74 nm to 156.04 ± 1.83 nm, an elevation in PDI from 0.19 ± 0.01 to 0.29 ± 0.02 over a 30 d storage period, and a reduction in zeta potential from −25.05 ± 2.07 mV to −17.35 ± 1.15 mV. The enlargement of CO-NLP primarily stemmed from their nanoliposomal nature, coupled with their larger surface area and the resultant high surface energy, leading to unstable energy levels. Consequently, CO-NLP became susceptible to the clumping of particles, resulting in an increase in the size of liposomes. This view could also be found in the study of Wang et al. [19]. These results indicate reduced stability of nanoliposomes at 25 °C compared to 4 °C. As depicted in Figure 4, microalgae oil stored at 4 °C exhibited less color discoloration and particle aggregation compared to storage at 25 °C.

#### 3.2.4. In Vitro Release Studies of Microalgal Nanoliposomes

**Figure 5:**
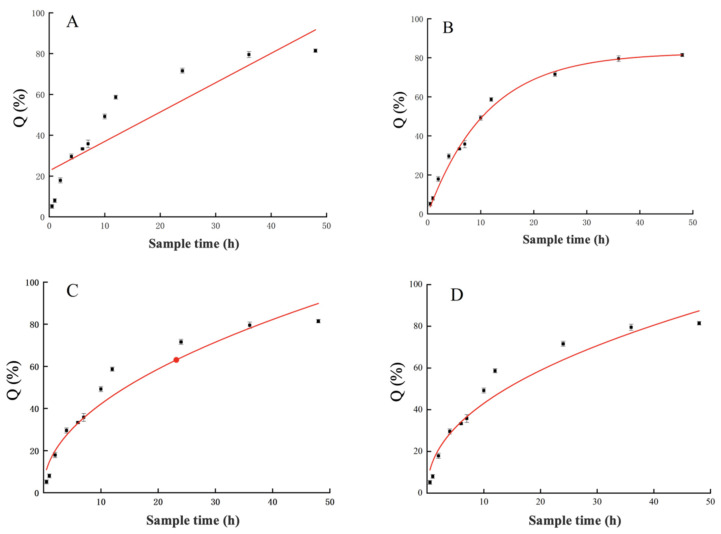
The in vitro release profiles of CO-NLP were determined by fitting four different release equations. (**A**) zero-level kinetic equation; (**B**) first-level kinetic equation; (**C**) Higuchi plane diffusion equation; (**D**) Retger-Peppas equation.

Due to the insolubility of chlorella oil in water, creating the necessary leaky conditions for in vitro release proves challenging. Therefore, this study utilizes Tween-80 as a solubilizing agent to augment the solubility of chlorella oil in PBS buffer, effectively addressing the challenge of leaky conditions. Figure 5 illustrates the in vitro release curves of CO-NLP, employing sampling time (t) and cumulative release percentage (Q) as the horizontal and vertical coordinates, respectively. Initially, the release rate of chlorella oil exhibited an ascending trend followed by a subsequent plateau, with the release rate of CO-NLP stabilizing at 33.35 ± 0.36% within the initial 6 h. After 6 h, the release rate of chlorella oil liposomes gradually increased until 48 h, achieving a cumulative release rate of 81.46 ± 0.83%. This indicates a noticeable slow-release effect of CO-NLP as a carrier of chlorella oil.

For a comprehensive understanding of chlorella oil release, Table 3 presents the fitted equations and correlation coefficients (R^2^) of various models derived from the zero-order kinetic equation, the first-order kinetic equation, the Higuchi plane diffusion equation, and the Retger-Peppas equation. The correlation coefficient for CO-NLP, as displayed in Table 3, was Q = 82.6282 (1 − exp (−0.0897t)), with an R^2^ value up to 0.9911, signifying the highest correlation coefficient. This aligns with Xiao’s [20] suggestion that the first-order kinetic model is more suitable for elucidating drug release. The hydrophobicity of the encapsulated material and the cohesiveness and continuity of the liposome membrane significantly influence the release behavior of liposomes [21].

#### 3.2.5. Bioavailability of CO-NLP

**Figure 6:**
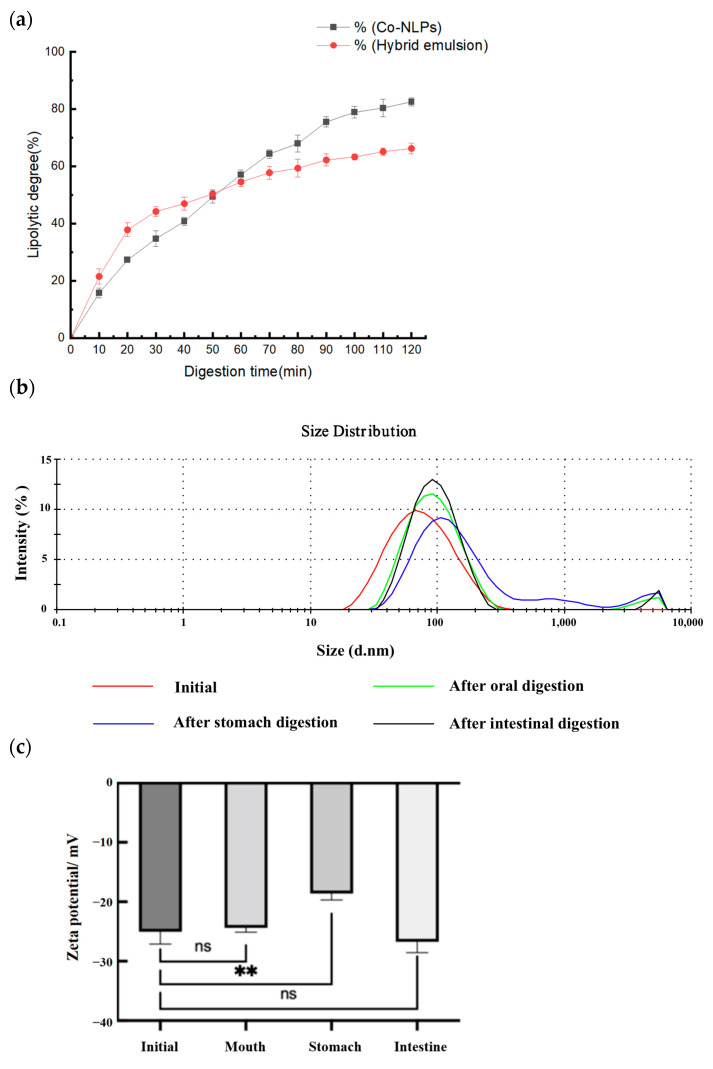
Assessment of the bioavailability and digestive properties of CO-NLP. (**a**) Kinetic curves illustrating the lipolysis of CO-NLP, Where the black curve represents the CO-NLP sample and the red curve represents the simple physical mixing emulsion made of microalgae oil and the embedding material; (**b**) Particle size of chlorella oil liposomes during digestion, among them, the red curve represents the undigested samples, the green curve represents the samples obtained after oral simulation digestion, the blue curve represents the samples obtained after gastric simulation digestion, and the black curve represents the samples obtained after intestinal simulation digestion; (**c**) Zeta potential changes during simulated digestion, where the ns refers to no significant difference between groups, while ** refers to significant difference between groups (*p* < 0.05).

Currently, numerous scholars are investigating the breakdown and assimilation of oil in the intestine by quantifying the release rate of unbound fatty acids during simulated intestinal digestion. In the presence of trypsin and bile salts, liposomes can undergo hydrolysis, liberating free fatty acids and reducing the pH of the environment. The lipolytic kinetic curve of chlorella oil liposomes is illustrated in Figure 6a, a consequence of the continuous addition of NaOH solution to neutralize free fatty acids and maintain the pH value of the digestion system constant at 7.0.

As depicted in the figure, the amount of free fatty acids released into the intestinal fluid environment steadily increased with the duration of digestion. The hydrolysis rate of CO-NLP experienced rapid escalation before stabilizing, and the release rate of free fatty acids from CO-NLP and the physically hybrid emulsion group (the formulation is the same as that of the nano-liposomes but the liposome structure is not formed) followed a similar trend. Within the initial 60 min of enteric digestion, the physically hybrid emulsion group exhibited a significantly higher release rate of free fatty acids compared to the CO-NLP group. However, after 60 min, the release rate of CO-NLP started to surpass that of the physically hybrid emulsion group. At the end of the simulated intestinal fluid digestion, the free fatty acid release rate of CO-NLP was 82.57 ± 1.45%, compared to 66.26 ± 1.87% in the hybrid emulsion group, indicating that CO-NLP is more proficient at undergoing lipolysis reactions in intestinal digestion.

The large specific surface area and weak spatial site resistance of nanotransport carriers, such as nanoliposomes, facilitate enhanced interaction between pancreatic lipase and encapsulated lipids, leading to a more efficient lipolytic reaction [26]. Additionally, the primary constituents of pancreatic enzymes in the small intestine—lipase, phospholipase A2, and cholesterol esterase—can break down lipids. Pancreatic lipase facilitates the hydrolysis reaction of fatty acids in phospholipids, resulting in the liberation of fatty acids and monoacyl lysophospholipids [27]. The disruption of the phospholipid emulsion layer structure by this digestion product leads to a further increase in the release rate of free fatty acids from liposomes [28]. Phospholipase A2 facilitates the breakdown of the sn-2 ester bond in phospholipids, resulting in the production of glycerophosphate and lysophospholipids; furthermore, cholesterol esterase, also referred to as bile salt-stimulating lipase, can hydrolyze phospholipids [29]. Additionally, bile salts have the ability to stimulate cholesterol enzymes, enabling the breakdown of lecithin and the formation of unbound fatty acids, resulting in a considerably elevated release rate of free fatty acids from CO-NLP compared to the control group in the later stages of digestion.

#### 3.2.6. Changes in Particle Size and Zeta Potential during Digestion of CO-NLP

The determination of particle size in the digestive product serves as a pivotal indicator of the digestive system, enabling the assessment of chlorella oil absorption status during the digestive process. Zeta potential, as a measure of the charge count on a particle’s surface, is commonly employed to signify the stability of a digestion system. Both particle size and zeta potential play a critical role in determining the physicochemical properties of the system throughout digestion. Figure 6b illustrates the mean particle size and zeta potential of the digestion products at various stages of digestion.

Subsequent to the in vitro digestion of CO-NLP, significant alterations manifested in their composition, structure, and stability. As depicted in Figure 6b, the treatment with simulated oral, simulated stomach, and simulated intestinal fluids induced fluctuations in the average particle size of CO-NLP. In the simulated oral phase, CO-NLP underwent flocculation, resulting in an increased particle size of 89.35 ± 1.38 nm. During the stomach phase, CO-NLP exhibited a higher flocculation rate, yielding an average particle size of 122.00 ± 7.81 nm for chlorella oil liposomes. Conversely, in the simulated intestinal phase, a reduction in particle size occurred, with an average size of 97.69 ± 0.60 nm for CO-NLP. The absorption of pepsin into the system transpires as CO-NLP traverses simulated gastric juice. The low pH in gastric juice, coupled with the absence of electrostatic repulsion or spatial site resistance, ensures system stability, causing aggregation and an increase in particle size [30]. CO-NLP and their encapsulations undergo hydrolysis by pancreatic lipase and bile salts in simulated intestinal fluid during the simulated intestinal phase. The resultant free fatty acids, bile salts, and phospholipids form a colloidal structure, potentially causing a decrease in the mean particle size of CO-NLP. Simultaneously, augmented electrostatic repulsion hinders oil droplet gathering, leading to a reduction in system particle size post-intestinal digestion [31]. The decrease in liposome particle size in the small intestinal environment may be attributed to the presence of bile salts, which possess surfactant properties that disrupt the phospholipid bilayer of liposomes [32].

Zeta potential is employed to calculate the surface charge of particles, and in the context of CO-NLP solution testing through in vitro digestion, alterations in zeta potential indicate a shift in the system’s interfacial makeup, as evident in Figure 6c. With the progression of digestion, there is a tendency for the zeta potential to decrease and then increase. Following simulated oral cavity digestion, a slight decline in the zeta potential of CO-NLP was observed, presumably influenced by the anionic element (mucin) present in saliva affecting the particles’ electrical characteristics [1]. After the simulated stomach stage, a significant decrease in the particles’ negative charge occurs due to the low pH and high ionic strength of the system post-digestion by simulated gastric juice. The acidic environment induces protonation of free fatty acids (-COOH), decreasing the overall charge on the particle’s surface, while strong ions create an electrostatic barrier [33]. The zeta potential experiences a substantial decrease due to gastric juice digestion. Finally, the zeta potential of CO-NLP in the simulated intestine phase increases to 26.73 ± 1.81 mV. Pancreatic lipase breakdown of microalgal oil produces anionic free fatty acids and monoglycerides, increasing the net charge of CO-NLP in simulated intestinal fluid. Consequently, the zeta potential exhibits a substantial increase in its absolute value [34]. Makino et al. [35] have demonstrated that the hydrophobic groups of phospholipids, when exposed to bile salts, can migrate to the surface of aqueous solutions, potentially causing an increase in the negative charge on the surface of liposomes after small intestinal digestion. Additionally, hydrolysis of phospholipids from liposome wall materials can lead to an increase in the negative charge.

### 3.3. Evaluation of the Lipid-Lowering Activity of CO-NLP

#### 3.3.1. Inhibition Activities of CO-NLP on Cholesterol Esterase

Recent scientific investigations have yielded compelling evidence supporting the medicinal properties of bioactive compounds derived from microalgae, suggesting their potential utility in addressing and preventing obesity [36]. In an illuminating study, Regueiras et al. [37] demonstrated the capacity of *Chlorella vulgaris* and *Chlorococcum amblystomatis* to modulate lipid metabolism in zebrafish larvae, concurrently mitigating steatosis in HepG2 liver cells burdened with excessive fatty acids. Furthermore, research by Yang and collaborators [38] revealed that Chlorella unsaturated fatty acids (C. UFAs), rich in linoleic acid, positively influenced body weight (resulting in a 13.93% reduction after 16 weeks of treatment), improved blood glucose levels (a 19.29% decrease), and enhanced lipid profiles (with a 23.45% reduction in triglycerides and an 8.76% decrease in total cholesterol) compared to C57BL/6J mice on a high-fat diet. This notable effect may be attributed to the reduction of hepatic lipid accumulation, achieved through the down-regulation of lipogenic genes (PPARγ, C/EBPα, LPL, aP2, FAS, and SREBP-1c) and the up-regulation of the lipolytic gene (adiponectin), representing a plausible underlying mechanism.

**Figure 7:**
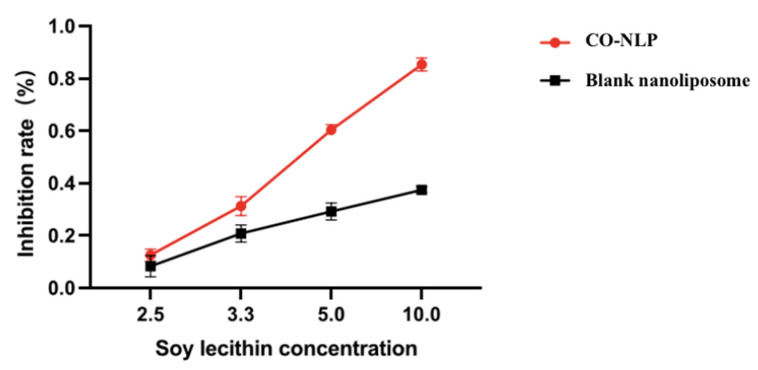
The inhibitory activity of the CO-NLP and the blank nanoliposome on the cholesterol esterase.

Pancreatic cholesterol esterase plays a pivotal role in the breakdown of dietary cholesterol esters via bile salt as well as the degradation of triglyceride-phospholipids, potentially impeding the absorption of dietary cholesterol [39]. The inhibition rate of CO-NLP on cholesterol esterase exhibited a dose-dependent nature, with a steady increase in inhibition rate corresponding to the rise in CO-NLP (Figure 7). Compared with blank nanoliposomes, the liposomes containing chlorella oil significantly increased the inhibitory activity of pancreatic cholesterol esterase, indicating the function of chlorella oil in lipid lowering.

#### 3.3.2. Assessment of the Hypolipidemic Impact In Vivo of CO-NLP in *C. elegans* MODEL

**Figure 8:**
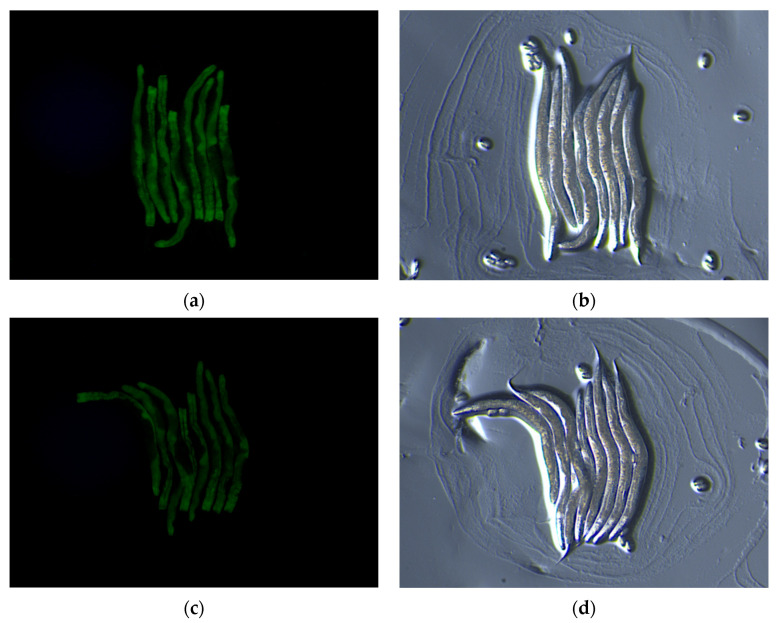
Representative images of dhs-3::gfp fluorescence in worms induced by blank nanoliposomes and CO-NLP. (**a**) Blank nanoliposome group (dark field, 100 px). (**b**) DMSO group (bright field, 100 px). (**c**) CO-NLP group (dark field). (**d**) CO-NLP group (bright field).

*C. elegans* is a good model organism for lipid storage studies due to its possession of regulatory factors and metabolic pathways akin to those governing adipose deposition and related metabolic diseases in mammals. Numerous lipid deposition mutants have been generated in *C. elegans*, including the dhs-3::gfp mutant, which manifests green fluorescence on lipid droplet surfaces, facilitating the facile observation of changes in lipid deposition. Therefore, we employed dhs-3::gfp mutants to observe the impact of CO-NLP on lipid accumulation in *C. elegans*, with image analysis performed using Image J. The results depicted in Figure 8 indicate that the mean fluorescence intensity of the blank nanoliposome group was 2.540 ± 0.043, whereas that of the 500 μg/mL CO-NLP group was 1.22 ± 0.02 (*p* < 0.001), signifying a reduction of nearly 52.17%. This result suggests that CO-NLP attenuates lipid accumulation in *C. elegans*, implying a potential reduction in in vivo adipose deposition.

## 4. Conclusions

The inherent hydrophobic characteristics of chlorella oils, combined with their limited water solubility, impede their integration into the majority of water-based food and beverage products. However, the use of liposome encapsulation facilitates their uniform dispersion in water.

In this study, chlorella oil nanoliposomes (CO-NLP) were synthesized employing a method involving ethanol injection and ultrasonication. The CO-NLP demonstrated a phospholipid bilayer structure, boasting an encapsulation efficiency of 92.84 ± 1.14%, an average particle size of 86.90 ± 0.74 nm, and a PDI of 0.19 ± 0.01. TEM revealed that the CO-NLP exhibited a spherical morphology with consistent size, maintaining exceptional stability at 4 °C. In vitro release experiments revealed a gradual release effect of CO-NLP, aligning closely with the primary release kinetic model. Simulated in vitro digestion experiments underscored the heightened efficacy of CO-NLP in lipid breakdown during intestinal digestion. The inhibition rate of cholesterol esterase by CO-NLP was determined to be 85.42 ± 0.25% at a concentration of 500 μg/mL. Notably, the average fluorescence intensity of *C. elegans* in the CO-NLP group was 52.17% lower than that of the control group, indicating the superior hypolipidemic function of CO-NLP.

These findings provide a solid theoretical foundation for the development of hypolipidemic functional foods. Liposome technology holds promise for enhancing the stability of chlorella oil and augmenting its bioavailability through controlled release, thereby fostering the integration of chlorella oil into food applications. The advancements in this study offer crucial technical support for the practical utilization of CO-NLP, opening up a broad spectrum of potential applications.

## Figures and Tables

**Table 1:** The quantity of additive agents used to evaluate cholesterol esterase inhibitory activity.

Tube	Cholesterol Esterase Solution (μL)	CO-NLP (μL)	PNPB (μL)	Buffer (mL)
Blank 1 (A_1_)	50	-	10	1
Blank control 2 (A_2_)	-	-	10	1
Sample 3 (A_3_)	50	25	10	1
Sample control 4 (A_4_)	-	25	10	1

**Table 2:** The fatty acid composition of chlorella oil.

Composition		Relative Content
Octanoic acid	C8:0	0.01 ± 0.00%
Carpic acid	C10:0	0.03 ± 0.01%
Hendecanoic acid	C11:0	10.50 ± 0.27%
Lauric acid	C12:0	0.15 ± 0.05%
Tridecanoic acid	C13:0	0.02 ± 0.00%
Myristic acid	C14:0	11.50 ± 0.15%
Myristoleic acid	C14:1	0.02 ± 0.01%
Pentadecanoic acid	C15:0	1.25 ± 0.11%
Pentadecanoic acid	C15:1	0.04 ± 0.01%
Palmitic acid	C16:0	25.02 ± 0.74%
Palmitoleic acid	C16:1	11.34 ± 0.91%
cis-10-heptadecenoic acid	C17:0	6.81 ± 0.96%
Heptadecanoic acid	C17:1	0.13 ± 0.02%
Stearic acid	C18:0	1.02 ± 0.19%
Oleic acid	C18:1n9c	2.51 ± 0.48%
Linolelaidic acid	C18:2n6t	1.71 ± 0.12%
Linoleic acid	C18:2n6c	1.71 ± 0.11%
gamma linolenic acid	C18:3n6	0.84 ± 0.04%
arachidic acid	C20:0	0.31 ± 0.02%
11-Eicosenoic acid	C20:1n9	0.02 ± 0.00%
11,14-Eicosadienoicacid	C20:2	0.01 ± 0.00%
cis-8,11,14-Eicosatrienoic	C20:3n6	0.20 ± 0.03%
Arachidonic acid	C20:4n6	1.46 ± 0.08%
cis-11,14,17-Eicosatrienoic acid	C20:3n3	0.02 ± 0.00%
Eicosapentaenoic acid (EPA)	C20:5	12.52 ± 0.19%
Heneicosanoic acid	C21:0	0.02 ± 0.00%
Docosanoic acid	C22:0	0.12 ± 0.02%
Erucic acid	C22:1n9	0.07 ± 0.01%
cis-13,16-Docosadienoic acid	C22:2	0.01 ± 0.00%
Docosahexaenoic acid (DHA)	C22:6n3	9.10 ± 0.13%
Tricosanoic acid	C23:0	0.01 ± 0.00%
Tricosanoic acid	C24:0	0.59 ± 0.31%
Nervonic acid	C24:1n9	0.94 ± 0.17%

**Table 3:** There are four kinds of release kinetic models.

Fitted Equation	Fitted Results	R^2^
Zero-level kinetic equation [22]	Q = 1.4402t + 22.5620	0.7764
The first-level kinetic equation [23]	Q = 82.6282(1 − exp(−0.0897t))	0.9911
Higuchi plane diffusion equation [24]	Q = 12.6950t^1/2^ + 1.9597	0.9351
Retger-Peppas equation [25]	Q = 15.20t^0.452^	0.9438

## Data Availability

Data is contained within the article and Supplementary Materials.

## Supplementary Materials

The following supporting information about the preparation technology of chlorella oil nanoliposomes can be downloaded: https://www.mdpi.com/article/10.3390/foods13010158/s1, Figure S1: Effect of lecithin and β-sitosterol mass ratio on the EE (a), particle size, and PDI (b) of CO-NLP; Figure S2: Effect of lecithin and chlorella oil mass ratio on the EE (a), particle size, and PDI (b) of CO-NLP; Figure S3: Effect of Tween-80 amount added on the EE (a), particle size, and PDI (b) of CO-NLP; Figure S4: Effect of PBS buffer pH on the EE (a), size, and PDI (b) of CO-NLP; Figure S5: Effect of ultrasonic duration on the EE (a), size, and PDI (b) of CO-NLP; Figure S6: Effect of ultrasonic power on the EE (a), size, and PDI (b) of CO-NLP; Table S1: Plackett-Burman design matrix and corresponding results for CO-NLP encapsulation; Table S2: Analysis of variance for Plackett-Burman design investigating the impact of various process conditions on the encapsulation rate of CO-NLP; Table S3: Analysis of variance for the Plackett-Burman design examining the influence of different process conditions on the particle size of CO-NLP; Table S4: Design and results of the Box-Behnken experiment; Table S5: Design and results of the Box-Behnken experiment; Table S6: Analysis of variance for the developed regression equation; Table S7: Analysis of variance for the developed regression equation; Table S8: Verification of experimental results for CO-NLP.
