# Supplementary material for: Nanoliposome-Mediated Encapsulation of Chlorella Oil for the Development of a Controlled-Release Lipid-Lowering Formulation
Source: Foods. 2024 Jan 2;13(1):158. doi: 10.3390/foods13010158 (PMC10779123; doi:10.3390/foods13010158)
Supplement: Supplementary file 1 [file foods-13-00158-s001.zip › foods-2761913-supplementary.pdf]

foods

MDPI

Supplementary Materials
Preparation of chlorella oil nanoliposomes

S1. Effects of technological single-factors on encapsulation efficiency (EE), particle size and polymer dispersibility index (PDI) of CO-NLP

S1. 1 Effect of lecithin to β-sitosterol mass ratio

Under fixed conditions, where the lecithin concentration was 10 mg/mL, the lecithin to chlorella oil mass ratio was maintained at 5:1, Tween-80 constituted 20% of the lecithin mass, pH was set at 6.8, ultrasonic treatment spanned 6 min at 200 W power, the encapsulation efficiency (EE), particle size, and PDI of Chlorella oil nanoliposomes (CO-NLP) were assessed across varying mass ratios of lecithin to β-sitosterol (2:1, 4:1, 6:1, 8:1, and 10:1).

The results, as depicted in **Fig. S1**, revealed a discernible trend. Notably, as the lecithin to β-sitosterol mass ratio increased, the encapsulation efficiency of CO-NLP exhibited an initial ascent followed by a subsequent decline. At a lecithin to β-sitosterol mass ratio of 8:1, the encapsulation rate peaked at 93.99±0.03%. Concurrently, at a lecithin to β-sitosterol mass ratio of 4:1, the average particle size and polydispersion coefficient attained their respective minima. It is noteworthy that at a lecithin to β-sitosterol mass ratio of 8:1, the polydispersity index surpassed 0.30, indicating an uneven distribution of particle sizes. Therefore, the optimal ratio of soybean lecithin to β-sitosterol was identified to lie within the range of 4:1 to 6:1.

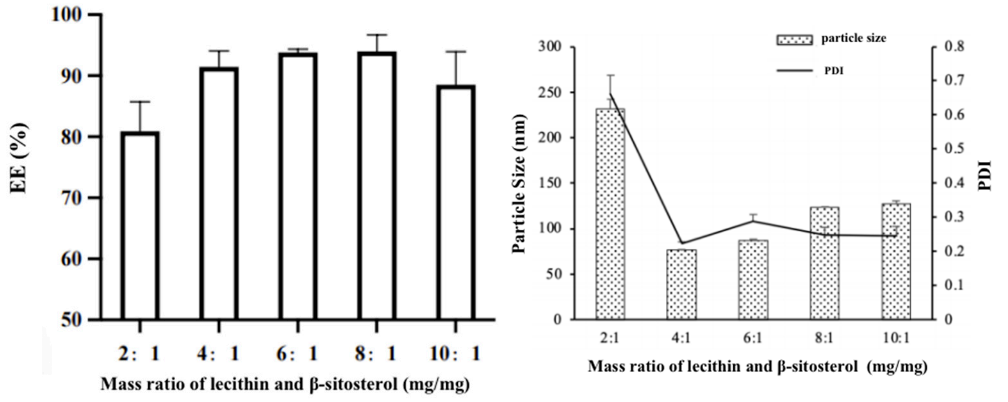

(a) (b)

Figure S1. Effect of lecithin and β-sitosterol mass ratio on the EE (a), particle size and PDI (b) of CO-NLP.

S1. 2 Effect of lecithin to chlorella oil mass ratio

Under standardized conditions featuring a lecithin concentration of 10 mg/mL, a fixed lecithin to β-sitosterol mass ratio of 5:1, the inclusion of Tween-80 at a proportion constituting 20% of the lecithin mass, a pH set at 6.8, ultrasonic treatment lasting 6 min with a power of 200 W, the encapsulation efficiency, particle size, and PDI of CO-NLP were scrutinized across varying mass ratios of lecithin to chlorella oil (2:1, 4:1, 6:1, 10:1, and 20:1).

The findings, elucidated in **Fig. S2**, revealed a discernible pattern. Evidently, as the mass ratio of lecithin to chlorella oil increased, the encapsulation rate of microalgal oil nanoliposomes exhibited an initial ascent followed by stabilization. Specifically, at a mass ratio of 4:1, the encapsulation rate plateaued, and at a mass ratio of 6:1, the liposome particle size and PDI values reached their nadir. Consequently, the appropriate range for the mass ratio of lecithin to chlorella oil was determined to be 5:1 to 7:1.

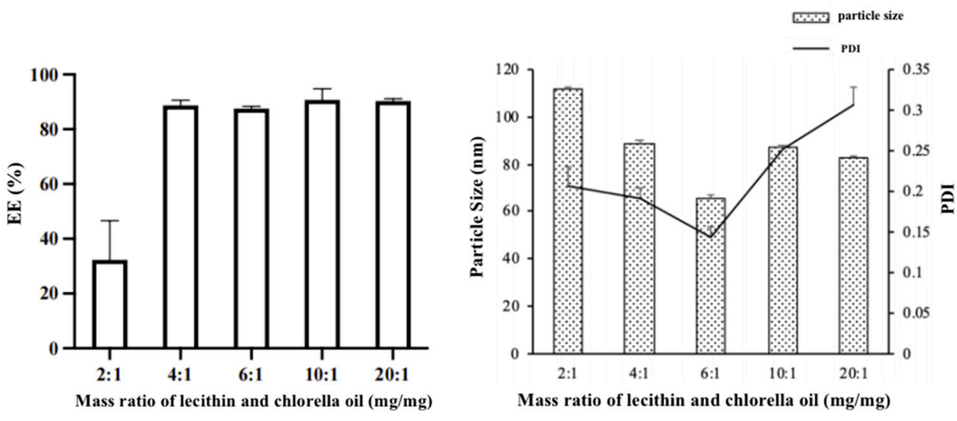

(a) (b)

Figure S2. Effect of lecithin and chlorella oil mass ratio on the EE (a), particle size and PDI (b) of CO-NLP.

S1. 3 Effect of different amounts of Tween-80

Under specified conditions involving a lecithin concentration of 10 mg/mL, a fixed lecithin to β-sitosterol mass ratio of 5:1, a lecithin to chlorella oil mass ratio of 5:1, a pH set at 6.8, ultrasonic treatment spanning 6 min at 200 W power, the encapsulation efficiency, particle size, and PDI of CO-NLP were assessed across varying addition amounts of Tween-80, accounting for 0%, 5%, 10%, 20%, and 30% of the lecithin mass.

The results, as depicted in **Fig. S3**, revealed distinct trends. Notably, as the addition amount of Tween-80 increased from 0% to 10%, the particle size of CO-NLP exhibited a gradual decrease, and the PDI value remained below 0.3, indicative of high stability. Conversely, when the addition amount of Tween-80 increased from 10% to 30%, all PDI values exceeded 0.3, indicating an uneven particle distribution, despite a continued decrease in particle size. In summary, the appropriate dosage range for Tween-80 in this experimental setup is deemed to be between 5% and 10%.

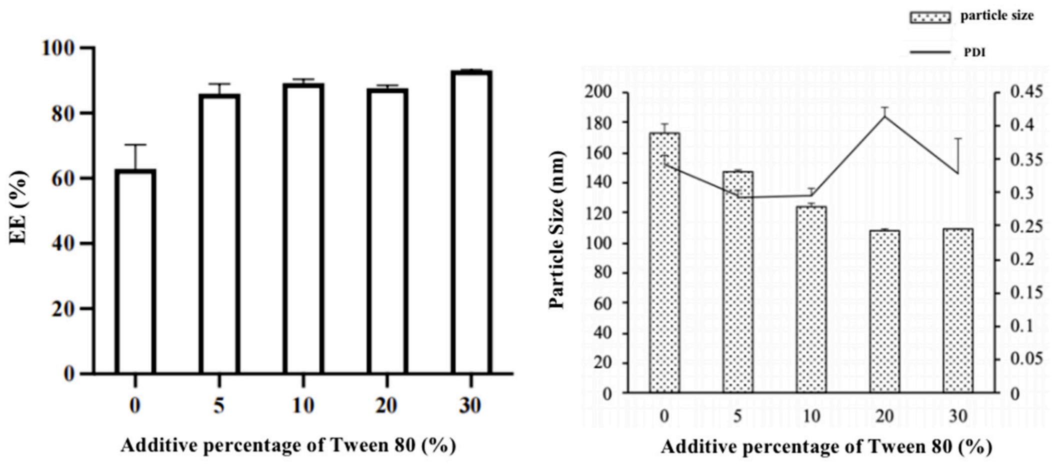

(a) (b)

Figure S3 Effect of Tween-80 amount added on the EE (a), particle size and PDI (b) of CO-NLP.

S1. 4 Effect of PBS buffer pH values

Under established conditions, featuring a lecithin concentration of 10 mg/mL, a fixed lecithin to β-sitosterol mass ratio of 5:1, a lecithin to chlorella oil mass ratio of 5:1, and a Tween-80 proportion of 20% relative to the lecithin mass, with ultrasonic treatment spanning 6 min at 200 W power, the encapsulation efficiency, particle size, and PDI of CO-NLP were evaluated across varying pH levels of the PBS buffer (6.0, 6.5, 7.0, 7.5, and 8.0).

The results, as illustrated in **Fig. S4**, delineated a discernible pattern. Evidently, as the pH of the PBS buffer increased, the entrapment rate of CO-NLP exhibited an initial ascent followed by a subsequent decline. Specifically, at a

pH of 7.0, the entrapment rate peaked at 91.10±0.002%, accompanied by diminutive particle size and PDI values. In summary, the judiciously selected pH range for the PBS buffer in this study is deemed to be 6.5-7.5.

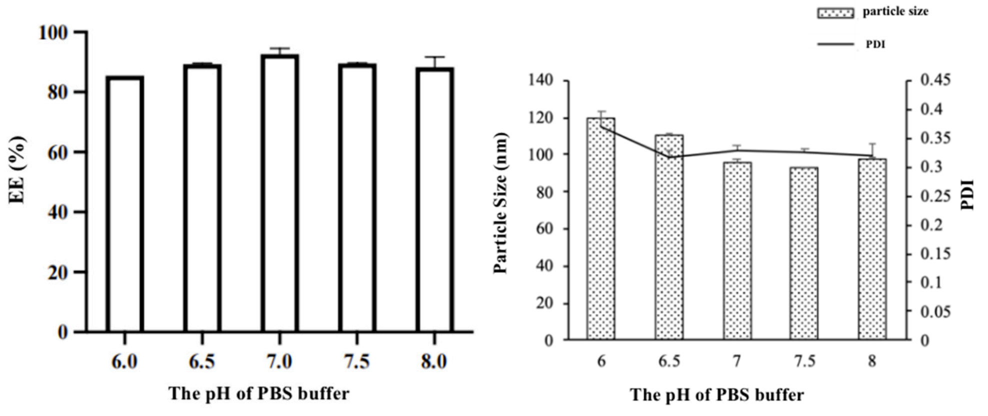

(a) (b)

Figure S4. Effect of PBS buffer pH on the EE (a), size and PDI (b) of CO-NLP.

S1. 5 Effect of ultrasonic duration

Under fixed conditions, with a lecithin concentration of 10 mg/mL, a lecithin to β-sitosterol mass ratio set at 5:1, a lecithin to chlorella oil mass ratio of 5:1, Tween-80 constituting 20% of the lecithin mass, and a pH of 6.8, the ultrasonic power at 200 W, the encapsulation efficiency, particle size, and PDI of CO-NLP were evaluated across varying ultrasonic treatment durations (0, 3, 6, 9, and 12 min).

The results are presented in **Fig. S5**. The findings revealed a noteworthy trend: the nanoliposome particle size exhibited a significant reduction ($P < 0.05$) following ultrasonic treatment. The encapsulation rate displayed an initial increase followed by a subsequent decline with the extension of ultrasonic time. This observed phenomenon is attributed to the extended ultrasonic exposure, which may compromise the integrity of the phospholipid bilayer membrane, leading to chlorella oil leakage and a subsequent reduction in encapsulation rate. Considering critical factors such as particle size, encapsulation rate, and process cost, the optimal range for ultrasonic treatment time was determined to be between 6 and 12 min.

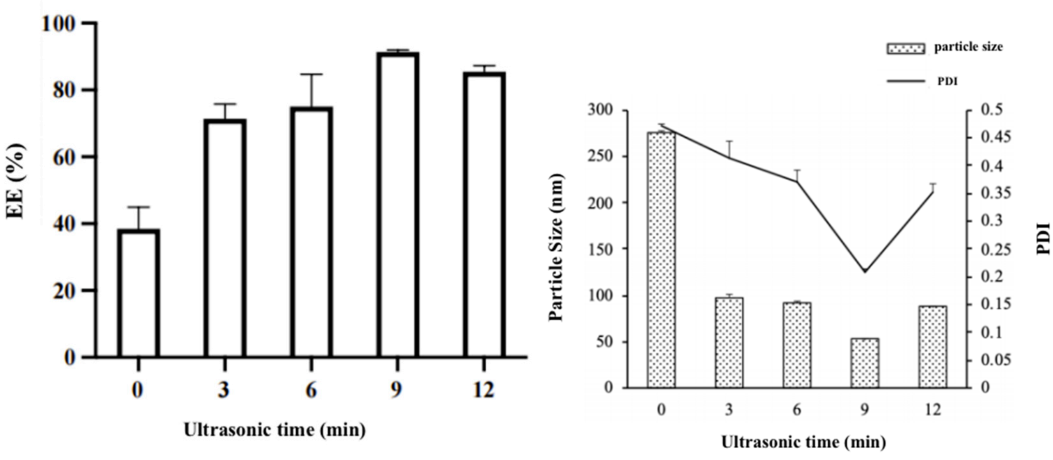

(a) (b)

Figure S5. Effect of ultrasonic duration on the EE (a), size and PDI (b) of CO-NLP.

S1. 6 Effect of ultrasonic power

Under standardized conditions, maintaining a lecithin concentration of 10 mg/mL, a lecithin to β-sitosterol mass ratio of 5:1, a lecithin to chlorella oil mass ratio of 5:1, with Tween-80 at 20% of the lecithin mass, at a pH of 6.8, and a fixed ultrasonic time of 6 min, the encapsulation efficiency, particle size, and PDI of CO-NLP were investigated across varying ultrasonic powers (100, 150, 200, 300, and 400 W).

The results are presented in **Fig. S6.** The findings demonstrated a discernible trend: the encapsulation efficiency of CO-NLP exhibited an initial increase followed by a subsequent decrease with the elevation of ultrasonic power. Under equivalent treatment time and at an ultrasonic power of 200 W, nanoliposomes displayed an average particle size of 91.49±1.66 nm, coupled with an encapsulation rate of 97.11±0.01%. However, excessively high ultrasonic power can elevate temperatures near the ultrasonic probe, reaching the phospholipid phase transition temperature and causing chlorella oil leakage, consequently reducing the encapsulation rate. In consideration of critical factors including particle size, encapsulation rate, and process cost, the optimal range for ultrasonic power selection was determined to be between 200 and 300 W.

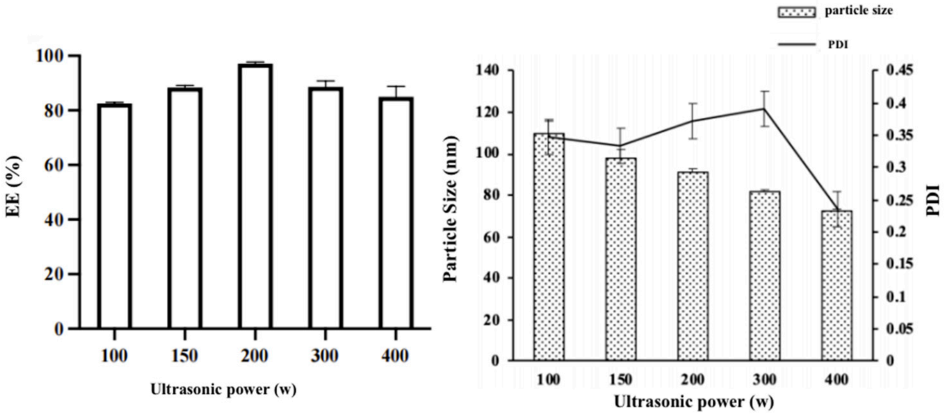

(a) (b)

Figure S6. Effect of ultrasonic power on the EE (a), size and PDI (b) of CO-NLP.

S2. Plackett-Burman test design for key process factor screening

Utilizing single-factor experiments as a foundation, the Plackett-Burman experiment was devised using Design Expert 8.0.6 software. This investigation delved into the significant impacts of various factors, namely, the mass ratio of lecithin to β-sitosterol (A), mass ratio of lecithin to chlorella oil (B), addition amount of Tween-80 (C), pH of PBS buffer (D), ultrasonic time (E), and ultrasonic power (F), on both the encapsulation rate and particle size of CO-NLP. A comprehensive presentation of factor values and outcomes is provided in **Table S1**. Further, statistical analyses of variance for the effects of distinct process conditions on encapsulation rate and particle size are detailed in **Tables S2 and S3**, respectively.

Table S1. Plackett-Burman design matrix and corresponding results for CO-NLP encapsulation.

Serial Number	A (mg/mg)	B (mg/mg)	C (%)	D	E (min)	F (W)	EE (%)	Particle Size (nm)
1	6：1	7：1	5	7.5	12	300	86.59	91.697
2	6：1	7：1	5	6.5	6	300	84.93	118.467
3	4：1	5：1	5	7.5	6	300	76.28	111.833
4	4：1	7：1	10	6.5	12	300	87.53	96.787
5	6：1	5：1	5	6.5	12	200	89.22	128.233
6	6：1	7：1	10	6.5	6	200	94.33	112.767
7	4：1	7：1	10	7.5	6	200	90.38	118.167
8	4：1	7：1	5	7.5	12	200	80.07	116.833
9	4：1	5：1	10	6.5	12	300	84.42	97.737
10	4：1	5：1	5	6.5	6	200	82.63	131.500
11	6：1	5：1	10	7.5	6	300	85.62	98.100
12	6：1	5：1	10	7.5	12	200	92.91	111.833

Table S2. Analysis of variance for Plackett-Burman design investigating the impact of various process conditions on the encapsulation rate of CO-NLP.

Sources of variance	Sum of squares	Freedom	Mean square	F	P
Model	268.03	6	44.67	8.78	0.0153
Mass ratio of lecithin to β-sitosterol（A）	86.89	1	86.89	17.08	0.0091
Mass ratio of lecithin to microalgae oil（B）	13.55	1	13.55	2.66	0.1636
Addition amount of Tween-80（C）	104.84	1	104.84	20.61	0.0062
pH of PBS（D）	10.47	1	10.47	2.06	0.2108
Ultrasonic time（E）	3.60	1	3.60	0.71	0.4387
Ultrasonic power（F）	48.68	1	48.68	9.57	0.0270
Residual	25.43	5	5.09		
Total error	293.46	11			

Table S3. Analysis of variance for the Plackett-Burman design examining the influence of different process conditions on the particle size of CO-NLP.

Sources of variance	Sum of squares	Freedom	Mean square	F	P
Model	1611.90	6	268.65	8.01	0.0187
Mass ratio of lecithin to β-sitosterol（A）	11.52	1	11.52	0.35	0.5831
Mass ratio of lecithin to microalgae oil（B）	50.10	1	50.10	1.49	0.2760
Addition amount of Tween-80（C）	332.57	1	332.57	9.92	0.0254
pH of PBS（D）	114.25	1	114.25	3.41	0.1242
Ultrasonic time（E）	189.71	1	189.71	5.66	0.0633
Ultrasonic power（F）	913.74	1	913.74	27.25	0.0034
Residual	167.63	5	33.53		
Total error	1779.53	11			

Upon scrutiny of Tables S2 and S3, it is evident that the P value for the encapsulation rate model is 0.0153 ($P < 0.05$), and for the particle size model is 0.0187 ($P < 0.05$), underscoring the significance of the relationship between the two regression equations. The adjusted correlation coefficients R2Adj for both models stand at 0.8094 and 0.7928, respectively, signifying robust regression with an explanatory capacity of approximately 80% of the response value variation. The models, thus, exhibit commendable fitting degrees.

The results highlight the substantive impact of the mass ratio of lecithin to β-sitosterol, the addition amount of Tween-80, and ultrasonic power on the encapsulation rate of nanoliposomes ($P < 0.05$). Similarly, the addition amount of Tween-80 and ultrasonic power significantly influence the particle size of nanoliposomes ($P < 0.05$). Conversely, the mass ratio of lecithin to chlorella oil, pH of PBS buffer, and average ultrasonic time exhibit no significant effects on the particle size distribution and encapsulation rate of microalgae lipids ($P > 0.05$).

Consequently, in this study, the mass ratio of lecithin to chlorella oil was held constant at 6:1, the pH of PBS buffer at 7.0, and the ultrasonic time at 9 min. However, the mass ratio range of lecithin to β-sitosterol (4:1~6:1), the addition amount range of Tween-80 (5~10%), and the ultrasonic power range (200~300 W) were further optimized utilizing the Box-Behnken response surface method.

S3. Optimization of key process parameters using the Box-Behnken response surface method

Drawing insights from both single-factor and Plackett-Burman experiments, the Box-Behnken response surface methodology was employed to optimize pivotal process parameters. The factors and levels of experimentation are documented in **Table S4**, while the experimental design and resultant responses are presented in **Table S5**. Variance statistical analyses for the influence of key process conditions on encapsulation rate (EE) and particle size, as derived from the Box-Behnken model, are provided in Tables S6 and S7, respectively. In **Table S6**, the P value for the encapsulation rate model is 0.0054 ($P<0.01$), and in **Table S7**, the P value for the particle size model is 0.0001 ($P<0.05$). These results affirm the significance of the relationship between the regression equations, demonstrating their applicability in capturing the intricate interplay between experimental independent variables and response values.

Table S4. Design of the Box-Behnken experiment.

Factor	Level		
	Low（-1）	Intermediate（0）	High（+1）
X1：Mass ratio of lecithin to β-sitosterol	4：1	5：1	6：1
X2：Addition amount of Tween-80（%）	5	8	10
X3：Ultrasonic power（w）	200	250	300

Table S5. Design and results of the Box-Behnken experiment.

Serial number	X1 (mg/mg)	X2 (%)	X3 (w)	EE (%)	Particle Size (nm)
1	6:1	7.5	200	89.56	112.60
2	4:1	7.5	300	83.74	99.12
3	5:1	7.5	250	92.25	97.14
4	5:1	5.0	300	91.45	106.10
5	6:1	5.0	250	88.73	107.20
6	5:1	7.5	250	93.34	92.40
7	4:1	5.0	250	81.42	103.01
8	5:1	5.0	200	92.57	119.60
9	4:1	7.5	200	86.26	107.31
10	6:1	7.5	300	92.57	101.40
11	5:1	7.5	250	91.45	92.77
12	5:1	10.0	200	93.34	112.90
13	5:1	10.0	300	92.25	104.70
14	5:1	7.5	250	95.22	95.18
15	5:1	7.5	250	93.71	93.45
16	4:1	10.0	250	88.48	104.70
17	6:1	10.0	250	91.97	98.75

Table S6. Analysis of variance for the developed regression equation.

Dependent variable	Sources of variance	Sum of squares	Freedom	Mean square	F	P
Y1：EE	Model	202.61	9	22.51	8.30	0.0054
	X1	65.72	1	65.72	24.23	0.0017
	X2	17.61	1	17.61	6.49	0.0382
	X3	0.3698	1	0.3698	0.1364	0.7228
	X1 X2	3.65	1	3.65	1.35	0.2841

	X1 X3	7.65	1	7.65	2.82	0.1370
	X2 X3	0.0002	1	0.0002	0.0001	0.9930
	$X1^2$	103.46	1	103.46	38.15	0.0005
	$X2^2$	1.45	1	1.45	0.5350	0.4883
	$X3^2$	0.1761	1	0.1761	0.0649	0.8062
	Residual	18.98	7	2.71		
	Misfitting term	10.66	3	3.55	1.71	0.3025
	Total pure deviation	8.32	4	2.08		
	Sum	221.59	16			

Table S7. Analysis of variance for the developed regression equation.

Dependent variable	Sources of variance	Sum of squares	Freedom	Mean square	F	P
Y2：Particle Size	Model	929.08	9	103.23	26.54	0.0001
	X1	4.22	1	4.22	1.08	0.3322
	X2	27.60	1	27.60	7.10	0.0323
	X3	211.05	1	211.05	54.27	0.0002
	X1 X2	25.70	1	25.70	6.61	0.0370
	X1 X3	2.27	1	2.27	0.5824	0.4703
	X2 X3	7.02	1	7.02	1.81	0.2209
	$X1^2$	12.96	1	12.96	3.33	0.1106
	$X2^2$	235.09	1	235.09	60.45	0.0001
	$X3^2$	353.65	1	353.65	90.94	< 0.0001
	Residual	27.22	7	3.89		
	Misfitting term	11.77	3	3.92	1.02	0.4735
	Total pure deviation	15.45	4	3.86		
	Sum	956.30	16			

According to the analysis results from Design-Expert 10.0.4 software, the optimal reference process conditions for CO-NLP preparation were determined. The recommended parameters include a mass ratio of lecithin to β-sitosterol at 5.26:1, an addition amount of Tween-80 at 8.18%, and an ultrasonic power of 264 W. The predicted encapsulation rate under these conditions was estimated at 93.92%, with an anticipated particle size of 92.69 nm. Validation of the optimized conditions was conducted through three experimental proof tests, as outlined in **Table S8**. The average encapsulation rate achieved was 92.84±1.14%, and the particle size measured at 86.90±0.74 nm. Comparative analysis with the predicted values revealed relative errors of 1.15% and 6.25%, respectively. These results signify the accuracy of both the encapsulation rate and particle size models in reflecting the impact of the three key preparation factors on CO-NLP.

Table S8. Verify experimental results.

Sirial number	EE（%）	Size（nm）	Zeta potential	PDI
1	94.03	87.34	-24.45	0.20
2	92.03	86.05	-23.32	0.19
3	93.63	87.31	-27.34	0.19
Average value	92.84±1.14	86.90±0.74	-25.05±2.07	0.19±0.01
